# Estimating the extra disability expenditures for the design of inclusive social protection policies

**DOI:** 10.3389/fresc.2023.1179213

**Published:** 2023-07-31

**Authors:** Daniel Mont

**Affiliations:** Center for Inclusive Policy, Washington, DC, United States

**Keywords:** disability, social protection, public policy, inclusion, social welfare

## Abstract

For social protection policies to be inclusive they must address the extra costs that people with disabilities incur. Studies show that these costs are highly significant and if not taken into account the economic wellbeing of people with disabilities is underestimated. Additionally, disability costs vary significantly by the type and degree of disability. To align the structure of social protection programs with how costs are incurred to promote equal participation requires estimating those costs. The Goods and Services Required approach, it is argued, is better than the often used Standard of Living Approach, and has implications for policy design.

## Introduction

Growing evidence suggests that people with disabilities are more likely to experience poverty than people without disabilities (1). Moreover, when multidimensional measures of poverty are used, as opposed to income or consumption measures, that difference is even greater (2, 3). This is because people with disabilities face a variety of barriers that prevent them from converting financial assets into desired life outcomes, or in the terms of Sen's and Nussbaum's capability approach, of moving from functionings to capabilities (4). Meeting their needs and overcoming those barriers entails additional expenditures.

Indeed, evidence suggests that people with disabilities incur substantial extra costs of living (5). These consist of both disability specific expenditures, such as those associated with assistive devices, personal assistance, and (re)habilitation, as well as increased spending on general items. People with disabilities often must spend more on transportation and health care, for instance, or on food for special diets (6). Even housing costs are affected, either because of needed household modifications to increase accessibility or because people must pay higher rents to live in newer construction or near workplaces, health care facilities, or accessible transportation.

One way to reduce these costs is to remove environmental barriers. If infrastructure and communication are more accessible, for example, the expenditures that families must make to overcome barriers to participation will be lower. Even with such efforts, people with disabilities will still incur extra costs to obtain the same standard of living as their nondisabled peers, especially in current environments where accessibility is often quite limited, and even more so in low and middle income countries.

If social protection programs do not account for these extra costs, they will fail people with disabilities in their goal of providing the level of well-being that they are designed to provide. Article 28 of the Convention on the Rights of Persons with Disabilities calls for an adequate standard of living. What is needed to maintain the same standard of living can be different for people with and without disabilities. Consider two households that are similar in every way (household size, area of residence, income, etc.) except one has a member with a disability and one does not. They both have similar needs for housing, food, clothing, and other necessities, but the household with a member with a disability has additional needs, for example assistive technology. They also have additional costs of undertaking basic activities – for example the need for special transportation or special food. Although they have the same level of income, they have different standards of living. Either they pay the extra costs associated with disability and have less for other necessities, or they do not cover those extra costs and thus undermine the wellbeing of the person with a disability. If a social protection program provides the same benefits to both households, it may raise the household without a disability over the poverty line, but still leave the household with a member with a disability effectively living below it.

The challenge is to adequately measure the extra expenditures necessitated by disability so that they can be incorporated into the design of social protection programs. Complicating this is the vast diversity of the population of people with disabilities. Disabilities vary significantly not only in the degree of disability, but also by the type of disability. People with vision difficulties face different costs than people with hearing difficulties, mobility difficulties, or complex medical needs (7). A one-size-fits-all approach will not address the diversity of costs (6, 8).

### Approaches to estimating the extra expenditures of disability

There are two basic approaches to measuring the expenditures associated with disability. The first is to measure what is currently being spent on goods and services that are attributable to the situation of people with disabilities. The second approach is to measure what goods and services are required for full participation. A gap may exist between what is spent and what is required for several reasons.

• Some goods and services required for participation may not be available where people live, especially if they live in a rural area in a low income country.
• Some people may not be aware of the types of goods and services that could allow them to participate more fully. Again, this is probably more common in low income countries, and among low income populations.
• Some households may be income constrained and not be able to afford all their basic necessities, and so must forego spending on some of them. That is, they can’t afford the things needed by their household members with disabilities and all other household necessities.
• Discrimination may exist within a household, and resources are kept from the person with a disability.

A common way of assessing the extra costs associated with disability is a method called the Standard of Living (SOL) method (9). This method measures the average extra expenditures households with a member with a disability are currently making to have the same standard of living as a household without a disability.

The basic idea of SOL is that two families with the same income and other characteristics are expected to have the same standard of living. If one of those households has a member with a disability, then any gap in wellbeing is assumed to result from the increased expenditures associated with the needs of the person with disability. The measure of the standard living could be an asset index but can also be other measures, such as a subjective measure of wellbeing.

A recent systematic review found that 18 studies covering 40 countries used the SOL estimated the extra costs of disability from 5.7% (amongst children with physical disabilities in the UK) to 155% of household income (adult 16 + with disabilities in Norway who are living alone) (10, 11). Costs varied widely by type and degree of disability (12–18).

SOL is a useful measure for examining the current economic impact of disability on households – and to what extent the current poverty line should be adjusted to get a better sense of how many households with disabled members are living below the effective consumption poverty line. However, it is not appropriate for adjusting social protection benefits for three reasons. First, it does not provide an estimate of the expense of goods and services required for full participation, which should be the goal of an inclusive social protection program. Second, it tends to provide lower estimates of the extra costs of disability in countries (generally poor countries) where the reasons for the gap between what is spent and what is required may be most pronounced. This last point is the finding a recent study using the SOL in a group of low income countries in Africa, where the estimated costs of disability were considerably lower than the costs estimated in higher income countries (19, 20).

Finally, the SOL method does not tell us what goods and services are needed. To that end, they can only inform us about the potential size of a cash benefit. It could be more efficient to meet the needs of people with different types of disabilities by targeting their specific needs, for example personal assistance and assistive technology. To do so, however, requires knowledge on how extensively they are needed and how much they cost.

**Figure 1:**
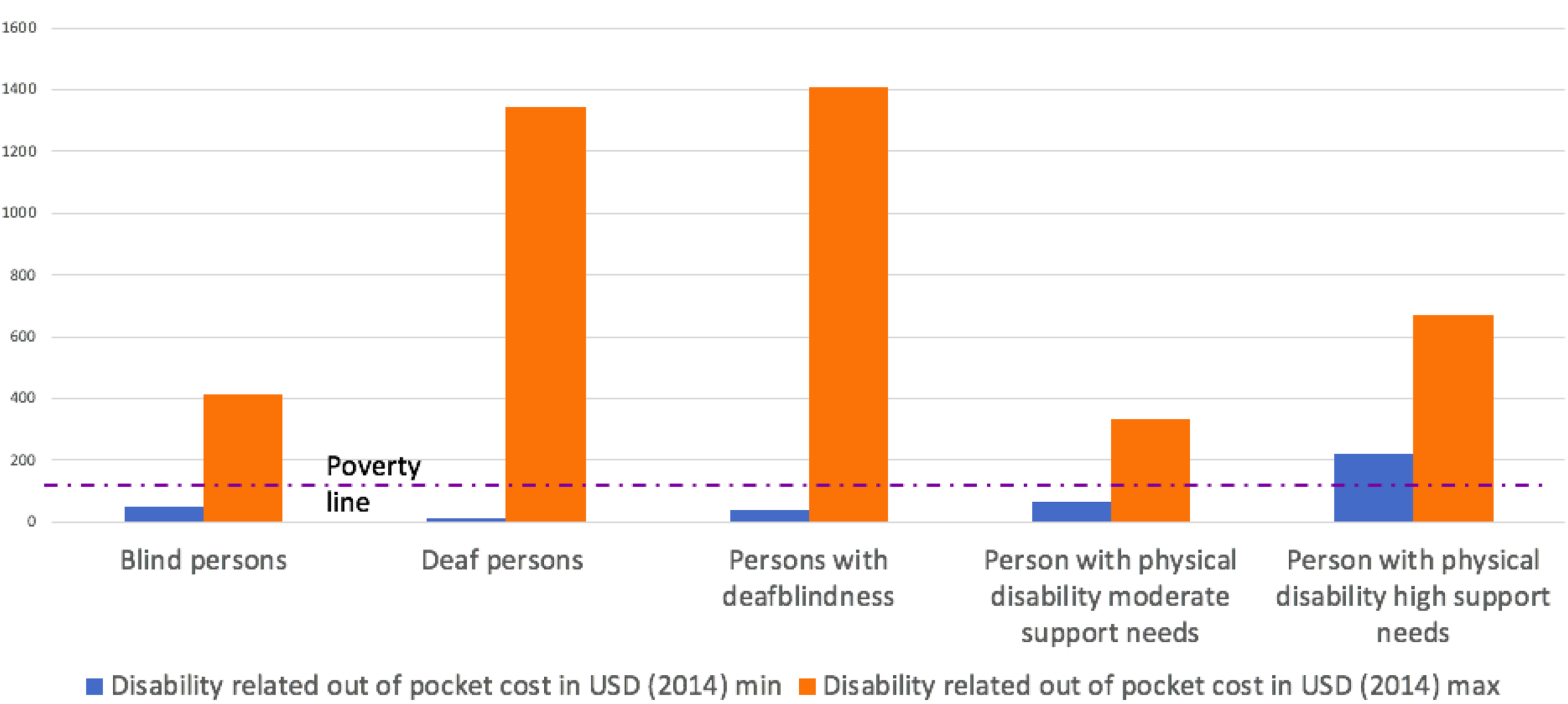
Minimum and Maximum disability related expenditures in South Africa, by type of disability. Source: Department of Social Development, Elements of the financial and economic costs of disability to households in South Africa. Results from a pilot study. 2015, DSD South Africa: Johannesburg.

When people are asked directly about what they are spending because of their disability, the range and nature of that spending varies dramatically. As can be seen in Figure 1, the range of expenditures both across and within types of disability is high. Providing a cash benefit equal to the average amount typically spent would not meet everyone's needs. Providing a cash benefit that would meet the needs of all people with disabilities would be highly inefficient.

The obvious questions are: What causes the high variance across disability types? And, what causes the high variance within disability types?.

**Figure 2:**
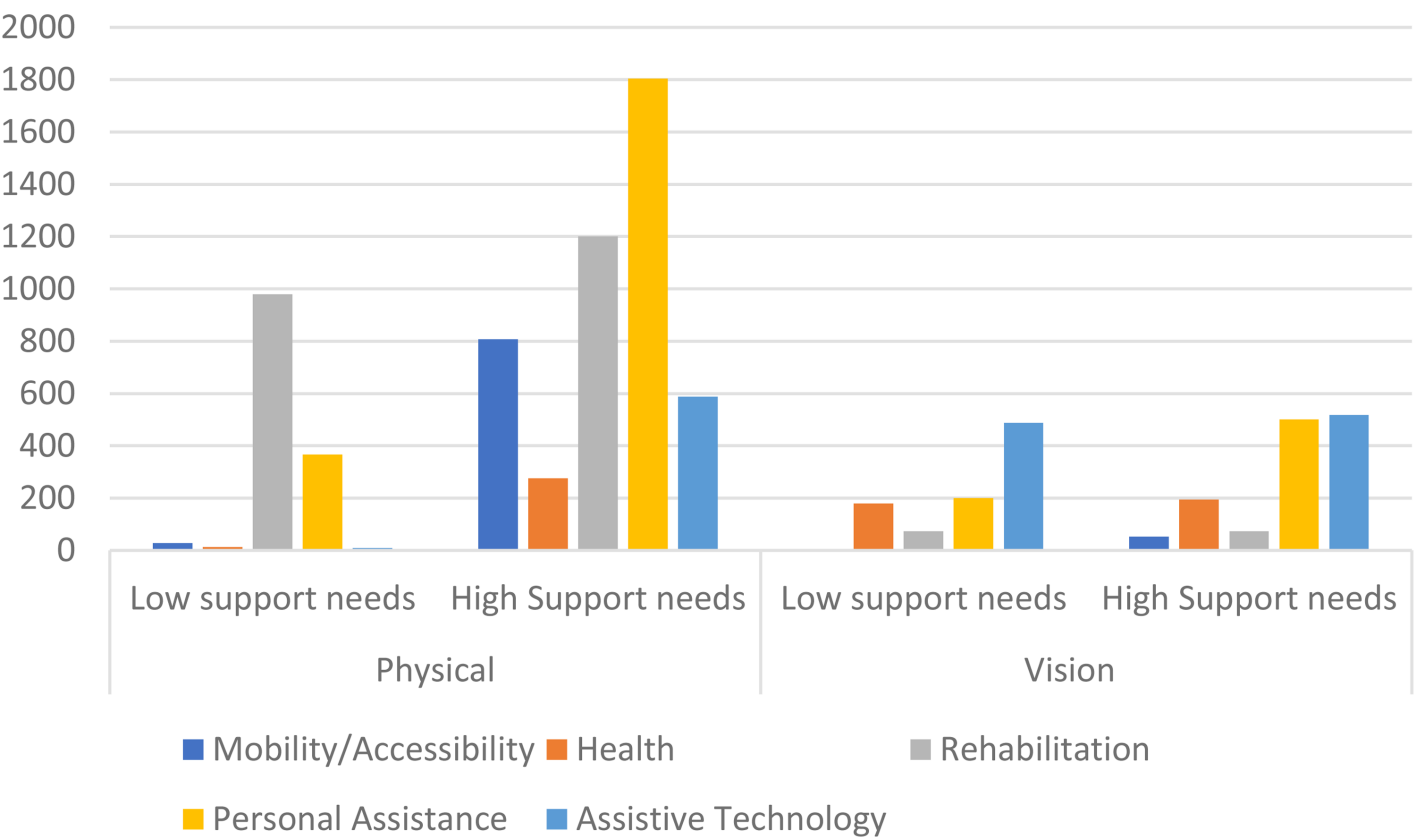
Extra monthly expenditures required for children with physical and vision disabilities by level of support needs. Source: UNICEF (2023) Goods and Services required for equal participation: Disability extra costs in Georgia.

The answer to the first question no doubt lies with the different types of goods and services needed. One answer to the second question (another will be discussed below) is that the degree of support needs might be different. Consider Figure 2 from a study of the costs of goods and services required for full participation of children with physical and vision disabilities in Georgia (21). Figure 2 shows the expenditures needed, by category, for children with each type of disability, and by degree of disability. Not only is the total amount needed very different by type of disability, and by degree of disability within each type, but so is the distribution across categories of support needs. For example, a child with physical disability and high support needs must spent 588 GEL per month (the Georgian currency) on assistive technology compared to only 119 GEL if they had low support needs. For children with visual disabilities those figures are 518 GEL and 487 GEL. Children with physical disabilities with high support needs must spend 1,805 GEL per month, over three times as much as a child with visual disabilities and high support needs.

Returning to Figure 1, we see that even among people who are deaf blind—and would all be considered in a high support needs category—the differences between the minimum and maximum expenditures are immense. The reason for this lies behind the difference between Figures 1, 2. Figure 1 shows the amount currently being spent. Figure 2 is an estimate of what is needed for full participation. As discussed earlier, there are reasons why there would be a gap between these two measures.

If a person is deaf-blind and is not participating in the community. That is, not going to school or to work or taking part in community life, they do not need to spend much. They can move about their home and communicate with their family. However, if they desire to participate in the social and economic life of their communities, they would require a full-time interpreter guide. That level of human assistance is expensive. They may choose not to participate exactly because of this expense. This effect may be especially pronounced among people who are deaf-blind, but it applies to people with all types of disability. Participating in society increases the expenditures associated with disability. One need not spend money on a modified vehicle or on taxis instead of public transportation if one is not venturing out of the house on a regular basis. This is truer when the barriers to participation present in the environment are greater. For example, one study from Turkey showed, using the SOL method, that extra expenditures are higher when people work (22).

The method for determining the disability costs—in amount and type—that are needed for equal participation is referred to as the Goods and Services Required (GSR) method (23). The GSR takes a mixed methods approach, drawing upon the expertise of people with disabilities, parents of children with disabilities, and service providers to arrive at a detailed description of the type and extent of specific goods and services required by people with various types of disabilities and level of support needs. This range of stakeholders is important for two reasons. First, these are the people with the best firsthand knowledge and experience of living with a disability in their country. Second, the participatory approach is important for building an understanding and acceptance of the methodology and the ensuing results.

After establishing an advisory group of key stakeholders to oversee the process, and an expert group to undertake the analysis, the expert group has several tasks. First, they must decide on the disability types they will consider and how to define low- and high support needs. Second, they must run focus groups to get wider input from the disability community on what goods and services are required. Then, they can make the initial estimates of the costs of required goods and services by type of disability and level of support needs. To finalize these estimates, they then conduct market research to fill in gaps where prices of needed goods and services may not exist. Early attempts at this method were done in South Africa (7, 24) and New Zealand (25) and were recently refined in a study of children with disability in Georgia (20). Projects are currently underway in Nigeria, Peru, and Tamil Nadu drawing upon the learnings of that approach.

### Incorporating disability costs in social protection schemes

One way to account for disability costs in social protection programs is to simply alter the threshold on means tests. This will make it easier for people with disabilities to qualify. However, as noted above, a means test adjustment pegged to the average costs of disability will be too large for some and too low for others, in particular for those with the greatest needs. If that adjustment is made based on SOL estimates, then it will not be based on the costs needed for participation and would probably be inadequate even for people with below the mean current extra expenditures. The same is true for a Guaranteed Annual Income (GAI) program, if the GAI level is increased for people with disabilities based on some estimate of current average extra expenditures. In fact, currently disability allowances tend to only support basic household consumption and not disability related costs (26).

Another approach is to consider the diversity of disability related costs that are needed for full participation. In fact, the best way to address these extra costs is to target the type of cost. The studies cited in this paper, for example, all point to the high costs of health care, personal assistance, assistive devices, and transportation. After supplying those in-kind, the variance of the remaining costs, across types and degrees of disability, is reduced substantially. They remain, but they are varied and idiosyncratic and could best be covered by a cash benefit.

The ultimate solution could be to offer a suite of programs offered in a progressive combination (6). These could include:

• *Health care including, including (re)habilitation and assistive devices*. People with disabilities often have extra medical expenses, and (re)habilitation and assistive devices can be essential for undertaking daily activities.
• *A cash allowance to cover the wide range of different types of disability related costs*. These include extra costs of general goods such as food, heat, and housing which people with disabilities often incur. This benefit could be provided at different levels depending on the severity of disability. Many countries already certify people with disabilities at different levels and adjust their benefits accordingly.
• *Human assistance* This would include personal assistance and sign language interpretation. It could be delivered via vouchers for purchasing services, a caregiver allowance to family members, or direct provision. The nature of the program could vary depending on the local context.
• *Concessions*. Subsidies could be provided for public transport, taxis, social housing or identified goods and services that are disproportionately needed by people with disabilities. A GSR study could help identify common items used by people with disabilities that could be subsidized.

Already in some high income countries, such as the United States, a suite of programs exists. But to ensure they fully cover the range and type of costs faced by people with different types and degrees of disabilities, without key gaps, a goods and services type study is needed.

And as noted above, along with these kinds of benefits—and to minimize the cost of providing them—investments should be made in creating a more accessible environment (physical and informational), improving health care systems, and making education systems more inclusive.

**Figure 3:**
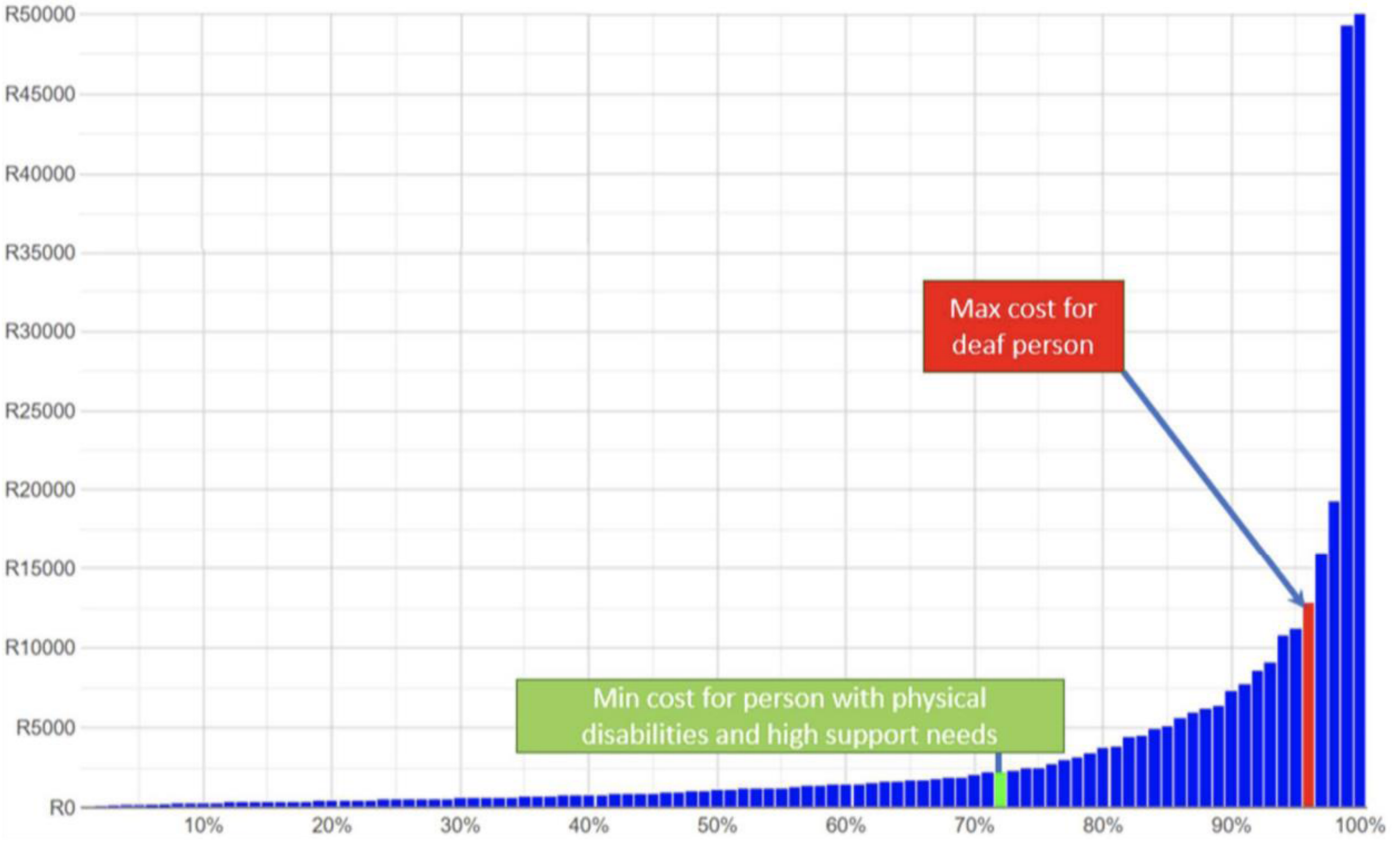
Income distribution in South Africa (2018) and estimates of disability expenditures (2015). Source: Mont, D, Cote, A, Hanass-Hancock, J, Banks, LM, Grigorus, V, Carraro, L, Morris, Z, and Pinilla-Roncancio, “Estimating the Extra Costs for Disability for Social Protection Programs,” UNPRPD, August 2022.

The question then arises about means testing for these benefits. However, in most low and middle income countries the ratio of extra costs to income is so high that means testing is not relevant. Figure 3 shows the distribution of income in South Africa compared to current expenditures by households related to their member with a disability. Those costs alone exceed the income of the large majority of the population. Requiring a means test on top of a disability certification does not appear warranted. In South Africa one would have to be in the top few centiles to be able to cover the extra costs observed and remain comfortably above the poverty line.

Moreover, it is important that disability benefits do not create a disincentive to work. Some countries, for example the United States, tie benefits to the inability to work (27). This is antithetical to the Convention on the Rights of Persons with Disabilities (28) in that it actively promotes non-participation, as noted in the Joint Statement on inclusive social protection, facilitated by the International Labor Organization and the International Disability Alliance (29). Requiring a means test to get benefits that are supposedly to help you participate—including working—is counterproductive.

## Conclusion

People with disabilities require additional expenditures to achieve the same opportunities for participation as their peers without disabilities and the same standard of living. If these are not accounted for, their level of wellbeing is being overestimated. For social protection programs to be inclusive they must account for these costs.

Simply estimating the average costs that people with disabilities are incurring is insufficient for the design of social protection programs. First, an average cost hides the great variance in costs faced by people with different types and degrees of disability. Some people incur very high costs, others’ costs are much lower. Second, it also hides the variance in the types of costs people incur. Some people have a high need for personal assistance, while others do not require it at all. Some people need assistive devices, while others do not.

To efficiently and adequately address disability costs of households, social protection programs must be structured in a way that aligns with those costs. One logical way to accomplish this is to develop a suite of programs that both target the specific types of costs that people face, while at the same time providing a cash benefit to cover idiosyncratic costs. Once the major categories driving the costs of disability are addressed, the residual can most likely be covered adequately by common cash benefits.

## Data Availability

The original contributions presented in the study are included in the article, further inquiries can be directed to the corresponding author.
